# Synaptic remodeling and the female depression exposome: a mini-review of neuroendocrine, epigenetic, and social determinants

**DOI:** 10.3389/fnins.2026.1783855

**Published:** 2026-04-07

**Authors:** Cindhi Mayra Rodrigues Xavier, Lucas Vinicius Faustino, Karina Maia Paiva, Rodrigo Freire Oliveira, Fausto Perdoná Guzen, José Rodolfo Lopes Paiva Cavalcanti, Dayane Pessoa de Araújo

**Affiliations:** 1 Multicenter Graduate Program in Physiological Sciences at the State University of Rio Grande do Norte, Mossoró, Brazil; 2 Postgraduate Program in Health and Society at the State University of Rio Grande do Norte, Mossoró, Brazil

**Keywords:** exposome, depression, epigenetics, synaptic remodeling, neuroendocrine aspects, women

## Abstract

Depression is a multifactorial, chronic disorder and represents a leading cause of disability, with women exhibiting nearly twice the lifetime prevalence compared to men. Growing evidence indicates that this disparity cannot be explained by hormonal or psychosocial factors, but rather by dynamic interactions between environmental exposures, neuroendocrine signaling, and epigenetic regulation across development. This mini-narrative review aimed to examine how sex-specific exposome components interact with epigenetic mechanisms and synaptic remodeling processes to influence vulnerability to Major Depressive Disorder in women. The reviewed evidence demonstrates that fluctuations in ovarian hormones modulate HPA axis responsivity, neuroinflammatory signaling, and glutamatergic transmission through epigenetic regulation of stress-responsive genes such as *NR3C1, SLC6A4*, and *BDNF,* consequently influencing synaptic remodeling within corticolimbic circuits. Environmental and social exposures, particularly early-life adversity and psychosocial stressors, further interact with microglial activation and chromatin remodeling to produce long-lasting alterations in hippocampal and prefrontal plasticity. Collectively, these findings support a model in which sex-dependent neuroendocrine sensitivity amplifies exposome-driven epigenetic programming across the lifespan. Future research directions emerging from this synthesis include longitudinal life-course studies integrating multi-omic biomarkers, quantitative exposome assessment, and neuroimaging approaches to identify modifiable environmental targets and advance precision, sex-informed preventive and therapeutic strategies in depression.

## 1 Introduction

Depressive disorders, including Major Depressive Disorder (MDD), affect approximately 300 million individuals worldwide and represent a leading cause of disability across the lifespan (Yuan et al., 2023). Early-life exposure to severe emotional stress or psychological adversity is a well-established risk factor for long-term impairments in biological functioning, neurodevelopment, and mental health (Yuan et al., 2023). Such adverse experiences increase vulnerability to depressive, anxiety-related, somatoform, and trauma-related conditions from childhood to adulthood. Notably, women exposed to early psychological adversity show a higher likelihood of developing emotional disturbances during pregnancy and the postpartum period, reinforcing the importance of sex-specific vulnerability trajectories. These epidemiological observations highlight the need to understand how environmental exposures are biologically embedded and translated into persistent neurobiological alterations (Yuan et al., 2023; Castro et al., 2024).

In this context, the exposome framework, introduced in 2005, refers to the cumulative environmental, lifestyle, and endogenous exposures experienced across the lifespan (Yuan et al., 2023). By encompassing psychosocial stressors, endocrine-disrupting chemicals, metabolic factors, and internal biological processes, the exposome highlights how external and internal environments interact dynamically with an individual’s chromosomal, hormonal, and physiological characteristics (Colwell et al., 2023). A key pathway through which these multilevel exposures exert lasting biological effects is epigenetic regulation. Epigenetic mechanisms act as a molecular interface capable of translating exposome-related inputs into stable or transient changes in gene expression without modifying the DNA sequence (Wang et al., 2023; Gutierrez-Ortiz et al., 2025). These mechanisms include DNA methylation, post-translational histone modifications (acetylation and methylation), and non-coding RNAs, which collectively regulate chromatin structure and transcriptional accessibility (Yuan et al., 2023; Lu et al., 2021). Growing evidence indicates that such epigenetic processes contribute to the pathophysiology of MDD and stress-related conditions by modulating neuroplasticity, monoaminergic neurotransmission, neuroinflammatory pathways, and HPA axis responsivity (Colwell et al., 2023; Wang et al., 2023; Dee et al., 2023).

The development of depressive disorders cannot be explained solely by genetic predisposition or simplified one-cause models, as early genomic approaches failed to account for the marked clinical heterogeneity observed in psychiatric conditions (Gutierrez-Ortiz et al., 2025). Instead, growing evidence indicates that depression arises from complex interactions among environmental exposures, neuroendocrine signaling and sex-specific biological factors. Differences in neurotransmitter systems, sex hormones such as estrogen and progesterone, and hypothalamic–pituitary–adrenal (HPA) axis regulation contribute to distinct vulnerability patterns between men and women (Wang et al., 2023). Environmental endocrine disruptors and stress-related exposures may further modulate neurodevelopment in a sex-dependent manner, particularly during sensitive developmental windows. Integrating exposome research with epigenetic and neuroendocrine perspectives may therefore clarify why women exhibit higher prevalence rates of depressive disorders after puberty and across reproductive transitions (Sikes-Keilp and Rubinow, 2021). In this mini-review, we examine how sex-specific epigenetic regulation mediates the relationship between lifelong environmental exposures and differential depression risk in women.

## 2 Methodology

All the procedures of the present mini-narrative review were performed in accordance with the Preferred Reporting Items for Systematic Reviews and Meta-Analyses (PRISMA) guideline. The search strategy was structured by combining Boolean operators and keywords directly related to the scope of the review, including: (exposome OR epigenetics) AND (depression OR major depressive disorder) AND (biological sex OR women OR men) AND (neuroendocrine OR synaptic mechanisms), allowing for the systematic identification of evidence on environmental, epigenetic, and sex-specific interactions associated with depression.

### 2.1 Inclusion criteria

**Figure 1:**
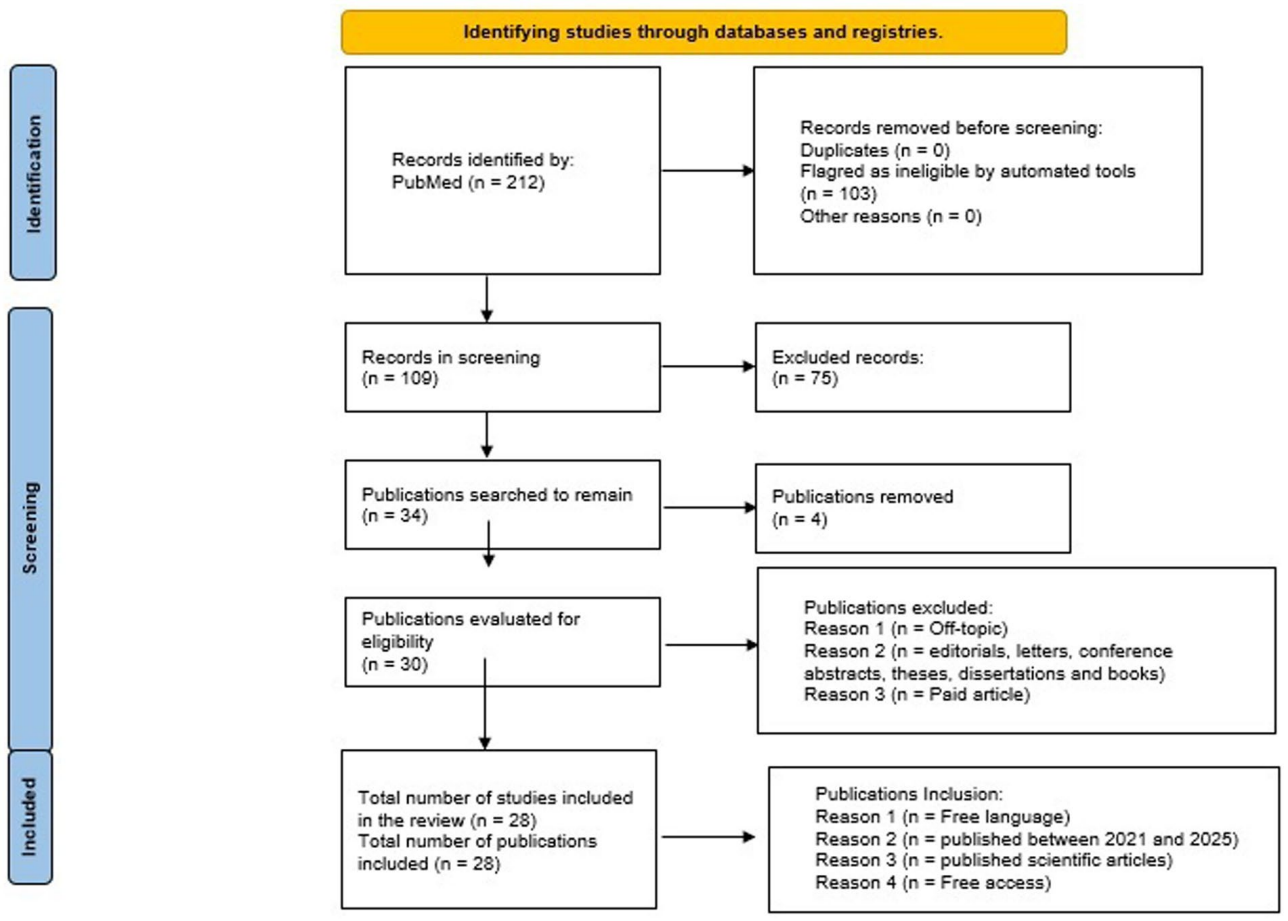
Flow diagram of the preferred reporting items for systematic reviews and meta-analyses (PRISMA) guideline applied to the mini-narrative review search strategy.

Scientific articles available in full and directly related to the topic were included, focusing on peer-reviewed articles published between January 2021 and December 2025. The keywords used were in English, and the articles found were in the same language. The inclusion criteria were as follows: (1) the study was a full-text article; (2) the criteria should address synaptic mechanisms and sex differences; and (3) studies including neurobiological markers in women as a primary or secondary analysis; (4) Studies based on *in vivo* or human experimentation involving biological women. Scientific articles published between 2021 and 2025 indexed in PubMed were considered during December 2025 (Figure 1).

### 2.2 Exclusion criteria

The exclusion criteria were as follows: (1) the effects of medications and the influence of pharmacology; and (2) the phenotype was not related to stress in studies focusing on gene–environment interaction, thus deviating from the topic. Another exclusion criterion was the exclusion of editorials, letters, conference abstracts, theses, dissertations, and books (Figure 1).

## 3 Genetic and molecular factors

Several stress-responsive genes exhibit reproducible in reproducible epigenetic alterations in humans and animal models following environmental adversity, including Nuclear Receptor Subfamily 3 Group C Member 1 (*NR3C1)*, oxytocin receptor (*OXTR)*, Solute Carrier Family 6 Member 4 (*SLC6A4*), and brain-derived neurotrophic factor *(BDNF*). Hypermethylation of the *NR3C1* promoter is associated with reduced glucocorticoid receptor expression, impairing HPA axis negative feedback and promoting sustained hypercortisolemia. Similarly, epigenetic modifications in *OXTR* and *SLC6A4* affect social bonding and serotonergic homeostasis, respectively, while reduced *BDNF* expression compromises neurotrophic support and adaptive plasticity. In parallel, altered histone modification patterns, characterized by decreased activating marks, like the Histone H3 Lysine 9 Acetylation (H3K9ac) and Histone H3 Lysine 14 Acetylation (H3K14ac), and increased repressive marks Trimethylation of histone H3 at lysine 27 (H3K27me3), promote transcriptional repression of genes involved in stress regulation and plasticity, frequently under chronic glucocorticoid signaling (Yuan et al., 2023; Dee et al., 2023). Together, these changes contribute to persistent neurobiological dysregulation in MDD (Schrott et al., 2022; Wei et al., 2022).

Sex-specific epigenetic signatures further modulate vulnerability to stress and depression. Experimental models demonstrate baseline differences in DNA methylation profiles between males and females, particularly in reward-related regions such as the nucleus accumbens. *DNMT3a* activity has been implicated in mediating stress susceptibility in a sex-dependent manner, influencing both transcriptional programs and behavioral outcomes (Hodes et al., 2024). These findings align with epidemiological data showing approximately twice the prevalence of MDD in women compared to men, suggesting that epigenetic regulation contributes to sexual dimorphism in stress responsivity and affective disorders (Schrott et al., 2022).

In women, fluctuations in estrogen and progesterone dynamically interact with epigenetic machinery and HPA axis function, influencing the expression of plasticity-related genes such as BDNF (Castro et al., 2024; Sikes-Keilp and Rubinow, 2021). Trauma exposure and psychosocial stressors are associated with sex-specific methylation patterns in stress and inflammation-related genes, including differential associations between *BDNF* methylation and perceived stress, as well as modulation of inflammatory mediators such as Interleukin-6 *(IL-6)* and Interleukin-8 (*IL-8)* (Hodes et al., 2024; Fungaro Rissatti et al., 2024). During pregnancy, maternal depressive symptoms and stress correlate with altered placental DNA methylation in pathways involved in neuronal migration, synaptogenesis, and cortical development, supporting a potential epigenetic mechanism underlying intergenerational transmission of vulnerability (Schrott et al., 2022; Tesfaye et al., 2021).

Importantly, these findings indicate that genetic susceptibility and epigenetic regulation do not operate as isolated mechanisms but are dynamically shaped by endocrine signaling across critical developmental and reproductive transitions (Kundakovic and Rocks, 2022). Hormonal fluctuations act as biological amplifiers capable of translating environmental exposures into long-lasting transcriptional and circuit-level adaptations, particularly within stress-responsive neural networks (Hodes et al., 2024; Luo et al., 2023). This convergence positions neuroendocrine regulation as a central mediator linking sex-specific epigenetic programming to behavioral outcomes, thereby providing a mechanistic framework for understanding female-biased depression risk and establishing the basis for examining neuroendocrine factors as key modulators of stress vulnerability across the lifespan (Schrott et al., 2022; de Assis et al., 2025).

## 4 Neuroendocrine factors

Gonadal steroids play a critical role in the formation, maturation, and maintenance of neuronal circuits across the lifespan by regulating *BDNF* expression and related neurotrophic pathways (Sikes-Keilp and Rubinow, 2021). *BDNF* mediated effects on neuronal proliferation, differentiation, and homeostasis are particularly relevant in regions implicated in affective regulation, including the prefrontal cortex (PFC), hippocampus, hypothalamus, amygdala, midbrain structures, and cerebellum (Sikes-Keilp and Rubinow, 2021; de Assis et al., 2025). Sex steroids modulate *BDNF* transcription both directly, via nuclear estrogen and progesterone receptors acting on genomic response elements, and indirectly, through intracellular signaling cascades that influence chromatin remodeling and transcriptional control (Schrott et al., 2022). Estradiol and progesterone may exert synergistic or competitive effects depending on receptor availability, coactivator recruitment, and activation of membrane-associated receptors, thereby dynamically shaping neurotrophic signaling in a context-dependent manner (de Assis et al., 2025).

The regulation of *BDNF* by sex steroids underscores the central role of ovarian hormones in synaptic plasticity, neuronal excitability, and circuit-level adaptation (Barzilay et al., 2021). Fluctuations in estradiol and progesterone during critical developmental and reproductive windows, including puberty, menstrual cycling, pregnancy, and the menopausal transition, modulate neural systems governing mood and stress responsivity (Hodes et al., 2024; Barzilay et al., 2021). Limbic and cortical regions such as the hippocampus, amygdala, anterior cingulate cortex, and PFC are consistently implicated in anxiety and depression-related phenotypes in both animal models and humans. Functional magnetic resonance imaging studies demonstrate cycle-dependent variability in hippocampal reactivity during emotional processing, with increased activation during the late follicular and mid-luteal phases compared to the early follicular and late luteal phases (Lu et al., 2021; Schrott et al., 2022). Estradiol levels positively correlate with hippocampal gray matter volume, white matter integrity, and task-related neural activity, supporting a structural–functional coupling between ovarian hormones and affective circuitry (Kundakovic and Rocks, 2022; Luo et al., 2023).

The onset of puberty represents a pivotal neuroendocrine transition associated with increased vulnerability to mood disorders in females (Lu et al., 2021). Following puberty, women exhibit approximately twice the prevalence of depression and suicidal ideation compared to men, reflecting the convergence of hormonal sensitivity and environmental exposures (Barzilay et al., 2021). Fluctuating estrogen and progesterone levels interact with both the hypothalamic–pituitary–gonadal (HPG) and HPA axes, modulating glucocorticoid responsivity and monoaminergic neurotransmission, including serotonergic, dopaminergic, and noradrenergic systems (Hodes et al., 2024; Luo et al., 2023; Barzilay et al., 2021). Periods of pronounced hormonal change, such as pregnancy and the perimenopausal transition, are also associated with transient reductions in hippocampal volume, followed by partial structural recovery postpartum or in postmenopause (Wei et al., 2022; Kundakovic and Rocks, 2022). These findings suggest that physiological ovarian hormone fluctuations can induce reversible structural remodeling in stress-sensitive regions, potentially contributing to sex-specific affective vulnerability (Hodes et al., 2024; Luo et al., 2023).

Sex differences in stress responsivity further involve differential HPA axis activation and inflammatory signaling (Sikes-Keilp and Rubinow, 2021). Women generally exhibit prolonged or amplified HPA activation in response to psychosocial stressors, which may interact with epigenetic regulation of stress and plasticity-related genes (Schrott et al., 2022). In contrast, men more frequently display externalizing phenotypes, including substance misuse and impulsive behavior, and higher lethality in suicide attempts, potentially reflecting sex-specific neuroimmune and behavioral adaptations (Fungaro Rissatti et al., 2024; Kundakovic and Rocks, 2022). Rather than representing isolated phenomena, these sex-dependent responses suggest that gonadal hormone fluctuations shape limbic–cortical circuitry, stress-axis regulation, and neurotrophic signaling in a sex-dependent manner across developmental windows (Schrott et al., 2022). Consequently, neuroendocrine dynamics can function as a critical interface, translating exposure to environmental stress into mechanisms of molecular plasticity and epigenetics that influence vulnerability to depression in women. It is important to emphasize that the nature, duration, and recurrence of these stressors are strictly determined by social inequalities, socioeconomic conditions, and environmental adversities, positioning social and contextual determinants as central factors in sex-specific risk trajectories, explored in the following section (Schrott et al., 2022; Luo et al., 2023; Takayanagi and Onaka, 2021).

## 5 Social, economic, and environmental factors

Environmental exposures during sensitive developmental windows, including prenatal life, early childhood, and adolescence, can durably increase vulnerability to psychiatric disorders through epigenetic and neurobiological programming. During these stages, neural circuits, as well as immune, endocrine and stress-response systems, undergo critical periods of maturation, rendering the brain particularly susceptible to environmental perturbations (Yuan et al., 2023; Erzin and Gülöksüz, 2021). Adversities such as psychosocial stress, toxic exposures, and infections may disrupt the developmental calibration of the HPA axis, immune signaling, and limbic–cortical connectivity, with long-term consequences for emotional regulation and stress responsivity (Erzin and Gülöksüz, 2021).

**Figure 2:**
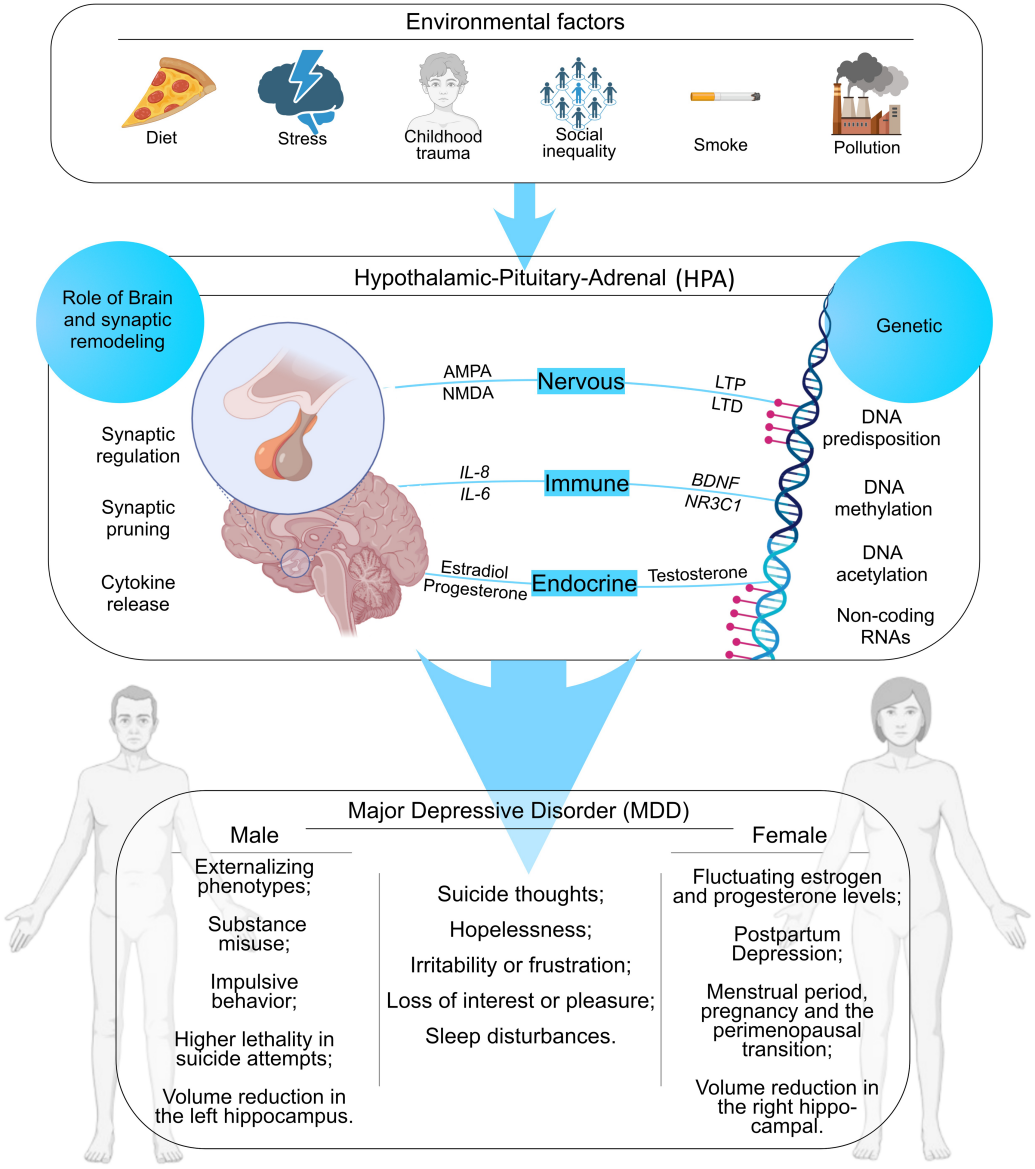
A set of epigenetic, hormonal, and environmental factors throughout life that can cause alterations in gene expression in genes related to cellular processes capable of predisposing to MDD in men and women. *α*-amino-3-hydroxy-5-methyl-4-isoxazolepropionic acid (AMPA), N-methyl-D-aspartate (NMDA), Long-Term Potentiation (LTP), Long-Term Depression (LTD), Interleukin-8 (*IL-8*), Interleukin-6 (*IL-6*), Brain-Derived Neurotrophic Factor (*BDNF*), and Nuclear Receptor Subfamily 3 Group C Member 1 (*NR3C1*). This image was created using the https://www.biorender.com/.

Evidence indicates that proximal social environments exert a stronger influence on depressive symptomatology than distal environmental factors (Yuan et al., 2023). Family dynamics, peer relationships, romantic involvement, and school-related stressors are consistently associated with the intensity and emergence of depressive symptoms, whereas factors such as air pollution or neighborhood green space show comparatively modest effects (Erzin and Gülöksüz, 2021; Deguen et al., 2022). Supportive and stable family environments exert protective effects by buffering stress reactivity, while chronic interpersonal conflict, relational dissatisfaction, and academic stress contribute to sustained HPA activation and inflammatory signaling (Erzin and Gülöksüz, 2021). These findings reinforce the relevance of the “social exposome” in shaping stress-related neural trajectories, particularly during periods of heightened neurodevelopmental plasticity (Deguen et al., 2022) (Figure 2).

Sex differences become particularly evident after puberty, when hormonal maturation interacts with cumulative environmental exposures (Lu et al., 2021; Barzilay et al., 2021). Epidemiological data demonstrate a marked increase in depression prevalence in females following pubertal onset, coinciding with fluctuations in ovarian hormones and increased exposure to gender-specific stressors (Hodes et al., 2024; Kundakovic and Rocks, 2022). Social inequalities and gender-specific stressors, including interpersonal violence, aesthetic pressures, economic insecurity, and role overload, disproportionately affect girls and women, promoting chronic activation of stress and inflammatory systems during periods of ongoing neural reorganization (Wang et al., 2023; Wei et al., 2022). In this context, sustained environmental burden interacts with heightened neuroendocrine sensitivity and epigenetic modulation of stress- and plasticity-related genes, amplifying limbic reactivity, disrupting circadian regulation, and increasing inflammatory responsivity (Luo et al., 2023). Consequently, the higher prevalence of depression in women after puberty reflects the dynamic convergence of cumulative external exposures and internal biological susceptibility rather than a single etiological determinant (Kundakovic and Rocks, 2022; Deguen et al., 2022).

Importantly, these convergent influences are biologically embedded through activity-dependent plasticity mechanisms that reshape neural connectivity across stress-sensitive circuits (Kundakovic and Rocks, 2022; Peña, 2026). Chronic psychosocial stress, hormonal fluctuations, and inflammatory signaling converge on molecular pathways regulating dendritic architecture, synaptic efficacy, and excitatory–inhibitory balance, suggesting that environmental adversity ultimately translates into structural and functional reorganization at the synaptic level (Luo et al., 2023; Deguen et al., 2022). This framework provides the mechanistic basis for examining synaptic remodeling as a central mediator linking social context to depression vulnerability in women (Fries et al., 2023).

## 6 Synaptic remodeling

Synaptic remodeling represents a central neurobiological interface through which environmental exposures, neuroendocrine signaling, and epigenetic regulation converge to shape emotional behavior across the lifespan. Structural plasticity encompasses dendritic arborization dynamics, synaptic turnover, and adult hippocampal neurogenesis, particularly within the dentate gyrus (Fries et al., 2023). Adaptive remodeling is promoted by environmental enrichment, physical activity, and learning-related stimulation, whereas chronic stress induces dendritic retraction and synaptic input reduction in stress-sensitive regions such as the hippocampus and medial prefrontal cortex (mPFC), impairing cognitive flexibility, emotional regulation, and memory consolidation. Circulating hormones, neurotransmitter systems, and neurotrophic factors collectively modulate these processes, positioning synaptic architecture as a primary substrate through which the exposome becomes biologically embedded within corticolimbic networks (Peña, 2026).

Epigenetic regulation directly influences synaptic plasticity by controlling chromatin accessibility and activity-dependent gene transcription. Experimental evidence demonstrates that histone deacetylase (HDAC) modulation alters the capacity of *BDNF* to enhance dendritic spine density and excitatory neurotransmitter release in hippocampal *Cornu Ammonis 1* (CA1) neurons, highlighting a functional coupling between chromatin remodeling and synaptic efficacy. Aging further interacts with stress-related epigenetic mechanisms, contributing to synapse loss, reduced adult neurogenesis, impaired long-term potentiation (LTP), and increased susceptibility to long-term depression (LTD) (Woodburn et al., 2021; Wang et al., 2024). Compared with younger individuals, aged prefrontal circuits show diminished structural recovery following chronic stress exposure, suggesting progressive loss of epigenetically mediated resilience mechanisms. Through activation of Tyrosine kinase B *(TrkB)* receptors and downstream MAPK/ERK, PLC–IP3, and mammalian target of rapamycin (mTOR) pathways, *BDNF* regulates axonal growth, dendritic pruning, protein synthesis, AMPA receptor trafficking, and glutamate release, thereby strengthening excitatory connectivity while newly generated adult-born neurons refine circuit integration (Bollinger et al., 2023; Patel et al., 2024; Gaspar et al., 2022).

Neuroimmune signaling further links environmental stressors to maladaptive circuit remodeling. Microglia integrate neuroendocrine signals, inflammatory mediators, monoaminergic activity, and gut–brain signaling to regulate synaptic pruning and spine turnover (Bollinger et al., 2023). Psychosocial stress and early-life adversity increase microglial activation within the hippocampus, amygdala, and hypothalamic paraventricular nucleus (PVN), promoting inflammatory signaling and excessive synapse elimination. In the prefrontal cortex, unpredictable chronic stress induces microglia-dependent synaptic pruning associated with Purinergic Receptor (*P2Y12)* receptor signaling, increased Colony Stimulating Factor 1 (*CSF-1)* expression, and astrocytic dystrophy characterized by reduced Glial Fibrillary Acidic Protein (*GFAP)* levels (Patel et al., 2024). Parallel hippocampal mechanisms amplify circuit vulnerability, as experimental *p21* overexpression in the dentate gyrus reproduces corticosterone-related phenotypes including reactive oxygen species accumulation, suppression of adult neurogenesis, dendritic atrophy, and anxiety-like behaviors (Peña, 2026). Together, these findings position neuroimmune activation as a mechanistic bridge through which psychosocial stress becomes structurally encoded within corticolimbic circuitry (Hakamata et al., 2022).

Glutamatergic signaling represents a central regulator of synaptic plasticity and emotional processing, acting as a primary interface between environmental stress exposure and circuit-level adaptation. MDD is consistently associated with region-specific glutamatergic dysregulation involving alterations in N-methyl-D-aspartate (NMDA) and *α*-amino-3-hydroxy-5-methyl-4-isoxazolepropionic acid (AMPA) mediated currents that disrupt excitatory inhibitory balance (Patel et al., 2024). Although dendritic spine loss in prefrontal pyramidal neurons may initially limit excitotoxic injury, sustained alterations ultimately impair executive function and learning processes. Pro-inflammatory cytokines modify receptor phosphorylation states and astrocytic glutamate clearance, increasing excitotoxic vulnerability while mitochondrial signaling regulates reactive oxygen species required for plasticity and dendritic spine elimination during LTD (Hakamata et al., 2022) (Figure 2).

Genetic findings further reinforce the synaptic basis of depression, as GWAS-associated genes such as Neuronal Growth Regulator (*NEGR1)* and Dopamine Receptor D2 (*DRD2)* regulate dendritic maturation, synaptic pruning, white matter integrity, and mTOR signaling pathways (Hakamata et al., 2022). Within limbic circuits, the hippocampus coordinates stress feedback through extensive connectivity with the prefrontal cortex, amygdala, hypothalamus, and thalamic nuclei, exhibiting sex-dependent vulnerability patterns characterized by lateralized volumetric reductions (Bollinger et al., 2023). Neuroendocrine transitions across puberty, menstrual cycling, pregnancy, and menopause modulate HPA axis responsiveness, rendering women particularly sensitive to early-life adversity and chronic stress exposure (Woodburn et al., 2021).

Some studies suggest that male patients may exhibit a more pronounced reduction in volume in the left hippocampus, while female patients may exhibit a more significant reduction in volume in the right hippocampus (Woodburn et al., 2021). Attenuated cortisol responses linked to exaggerated hippocampal or PVN feedback, combined with hippocampal gray matter reduction and amygdala hyperreactivity, may be perpetuated by epigenetic mechanisms across generations, contributing to sex-specific susceptibility to depressive phenotypes and differential responses to glutamatergic-targeted therapies (Bollinger et al., 2023). Taken together, these findings position synaptic remodeling as a central biological substrate through which genetic susceptibility, neuroendocrine transitions, and environmental exposures converge to shape sex-specific depression vulnerability trajectories (Woodburn et al., 2021; Wang et al., 2024). In women, repeated hormonal transitions and cumulative stress exposure can amplify changes in circuit plasticity throughout life, reinforcing synaptic remodeling as a key mechanistic interface linking exposure to affective pathology (Bollinger et al., 2023).

## 7 Conclusion

Collectively, the evidence reviewed indicates that sex-specific interactions between environmental exposures, neuroendocrine regulation, and epigenetic mechanisms converge to shape differential vulnerability to MDD across the lifespan. Rather than reflecting isolated biological determinants, depression risk appears to emerge from the dynamic integration of the exposome with sex-dependent hormonal transitions, stress responsivity, neuroimmune signaling, and synaptic plasticity pathways.

Current findings consistently demonstrate epigenetic modulation of stress-related genes and neuroplasticity processes; however, heterogeneity in study design, exposure characterization, and temporal assessment limits causal inference regarding how male and female exposomes differentially influence epigenetic programming. Addressing these limitations will require longitudinal life-course studies integrating multi-omic biomarkers and refined quantitative exposome assessment. These gaps highlight the need for integrative approaches capable of incorporating environmental, biological, and developmental variables, ultimately enabling the identification of modifiable exposures and the development of sex-informed preventive and therapeutic strategies.
